# An Ensemble-Based Deep Convolutional Neural Network for Computer-Aided Polyps Identification From Colonoscopy

**DOI:** 10.3389/fgene.2022.844391

**Published:** 2022-04-26

**Authors:** Pallabi Sharma, Bunil Kumar Balabantaray, Kangkana Bora, Saurav Mallik, Kunio Kasugai, Zhongming Zhao

**Affiliations:** ^1^ Department of Computer Science and Engineering, National Institute of Technology Meghalaya, Shillong, India; ^2^ Computer Science and Information Technology, Cotton University, Guwahati, India; ^3^ Center for Precision Health, School of Biomedical Informatics, The University of Texas Health Science Center at Houston, Houston, TX, United States; ^4^ Department of Gastroenterology, Aichi Medical University, Nagakute, Japan; ^5^ Human Genetics Center, School of Public Health, The University of Texas Health Science Center at Houston, Houston, TX, United States; ^6^ MD Anderson Cancer Center UTHealth Graduate School of Biomedical Sciences, Houston, TX, United States

**Keywords:** colorectal cancer, deep learning, polyp detection, colonoscopy, ensemble classifier

## Abstract

Colorectal cancer (CRC) is the third leading cause of cancer death globally. Early detection and removal of precancerous polyps can significantly reduce the chance of CRC patient death. Currently, the polyp detection rate mainly depends on the skill and expertise of gastroenterologists. Over time, unidentified polyps can develop into cancer. Machine learning has recently emerged as a powerful method in assisting clinical diagnosis. Several classification models have been proposed to identify polyps, but their performance has not been comparable to an expert endoscopist yet. Here, we propose a multiple classifier consultation strategy to create an effective and powerful classifier for polyp identification. This strategy benefits from recent findings that different classification models can better learn and extract various information within the image. Therefore, our Ensemble classifier can derive a more consequential decision than each individual classifier. The extracted combined information inherits the ResNet’s advantage of residual connection, while it also extracts objects when covered by occlusions through depth-wise separable convolution layer of the Xception model. Here, we applied our strategy to still frames extracted from a colonoscopy video. It outperformed other state-of-the-art techniques with a performance measure greater than 95% in each of the algorithm parameters. Our method will help researchers and gastroenterologists develop clinically applicable, computational-guided tools for colonoscopy screening. It may be extended to other clinical diagnoses that rely on image.

## 1 Introduction

Cancer is a complex disease caused by uncontrolled cell growth. Colorectal cancer (CRC) is a form of cancer that occurs when irregular growth occurs in the colon and rectum (the last part of the gastrointestinal (GI) system). A polyp’s initial stage is noncancerous; however, some polyps may become cancerous over time. For the determination of the treatment plan, the identification of a polyp is essential. Regular screening can prevent cancer through the identification and removal of precancerous polyps (Soh et al., 2018; Sánchez-Peralta et al., 2020). Diagnosis of the disease at an early stage can result in more effective treatment. As a consequence, screening decreases CRC mortality by both reducing the incidence and increasing survival. The visual test is a commonly recommended technique for CRC screening. Colonoscopy is one of the standard screening techniques for visualizing specific parts of the colon (Sánchez-Peralta et al., 2020). During a colonoscopy, gastroenterologists perform visual screening of the entire colon from the rectum to the cecum with the help of a light and tiny camera attached to the colonoscope.

Most of the works available in literature have focused on the detection of different types of polyps, such as cancerous or noncancerous, due to the lack of availability of a benchmark dataset. However, a colonoscopy video contains frames with polyps and without polyps. Therefore, as the first step, it is necessary to conduct a study to classify the frames to examine the presence of polyps, which will further study the features of the polyps, such as whether it is cancerous or not, location on the colorectum, or the disease stages.

Multiple computer-aided design approaches have been proposed in previous studies that can be applied to CRC analysis. In this direction, most of the works have used k-means, Fuzzy C-means, K-Nearest Neighbor (KNN), and support vector machine (SVM) based on handcrafted features (Häfner et al., 2015; Wimmer et al., 2016; Ševo et al., 2016; Shin and Balasingham, 2017; Sanchez-Gonzalez et al., 2018; Sundaram and Santhiyakumari, 2019). For example, Oh et al. (2007) used edge detection–based methods and achieved 96.5% accuracy in detecting informative frames. Recent studies have introduced the applicability of deep learning in colon cancer detection (Bernal et al., 2017; Pacal et al., 2020). Bernal et al. (2017) compared the efficacy of handcrafted features with CNN-extracted features in detecting polyp presence on still frames. They claimed that end-to-end learning approaches based on the CNN are more efficient than those based on handmade features. Akbari et al., (2018) applied the CNN on whole-slide images to classify informative and noninformative frames. Others (Ribeiro et al., 2016; Sharma et al., 2020a; Sharma et al., 2020b) also utilized deep learning architecture, such as VGG, ResNet, and GoogLeNet, for informative frame detection. Graham et al. (2019) used a minimum information loss deep neural network to segment the polyp region; they could achieve an F1 score of 0.825 and object-level dice score of 0.875. Sornapudi et al. (2019) proposed a CNN-based approach and used transfer learning from the ImageNet dataset to achieve an 88.28% F1 score in polyp segmentation.

The literature proffers a clear trend to eventually replace handcrafted features and traditional ML techniques with end-to-end frameworks. It all enables significant improvement in colonoscopy image analysis, making it more automated and providing more reliable and precise polyp detection methods. This work proposed a fully automatic system to classify polyps on still-frames from colonoscopy. The proposed system is an ensemble of different CNN architectures. The system will provide a decision in two stages. First, the frames are assessed as informative (frames containing polyps) and uninformative (frames not containing polyps). Second, the same classification model is applied to predict informative frames as cancerous (frames containing cancerous polyps) or noncancerous (frames containing non-cancerous polyps). Ensemble learning is an approach where better efficiency is obtained by integrating the results into one high-quality classifier from multiple classification models. Our methodology also addresses the problems involved in the use of the CNN for classification with limited sample data by using pre-trained CNN on a large dataset of natural images ( >1 million) and fine-tuning (optimizing) them using a smaller medical image dataset (at the thousand level). The different CNNs in our Ensemble method allow extracting the image features on different semantic levels so that the distinctive and subtle variations between different image classes can be identified. The contribution of this work includes the following three parts:

• Development of an automatic polyp detection model from colonoscopy images. Our model will classify the colonoscopy frames as informative or uninformative and further classify informative frames as cancerous or noncancerous.
• Detection of polyps during colonoscopy screening through the multiple classifier consultation strategy to create an effective and strong classifier for polyp identification. After our literature review, we assessed that it is the first approach by an Ensemble of various significant learning models for colonoscopy frame analysis.
• The robustness of the proposed Ensemble classifier is demonstrated by applying it to a real-world clinical dataset and comparing its result with the publicly available benchmark dataset. A suitable statistical significance test is conducted to assess the significant difference in the performance of proposed methods with a single classifier.

## 2 Materials and Methods

**FIGURE 1:**
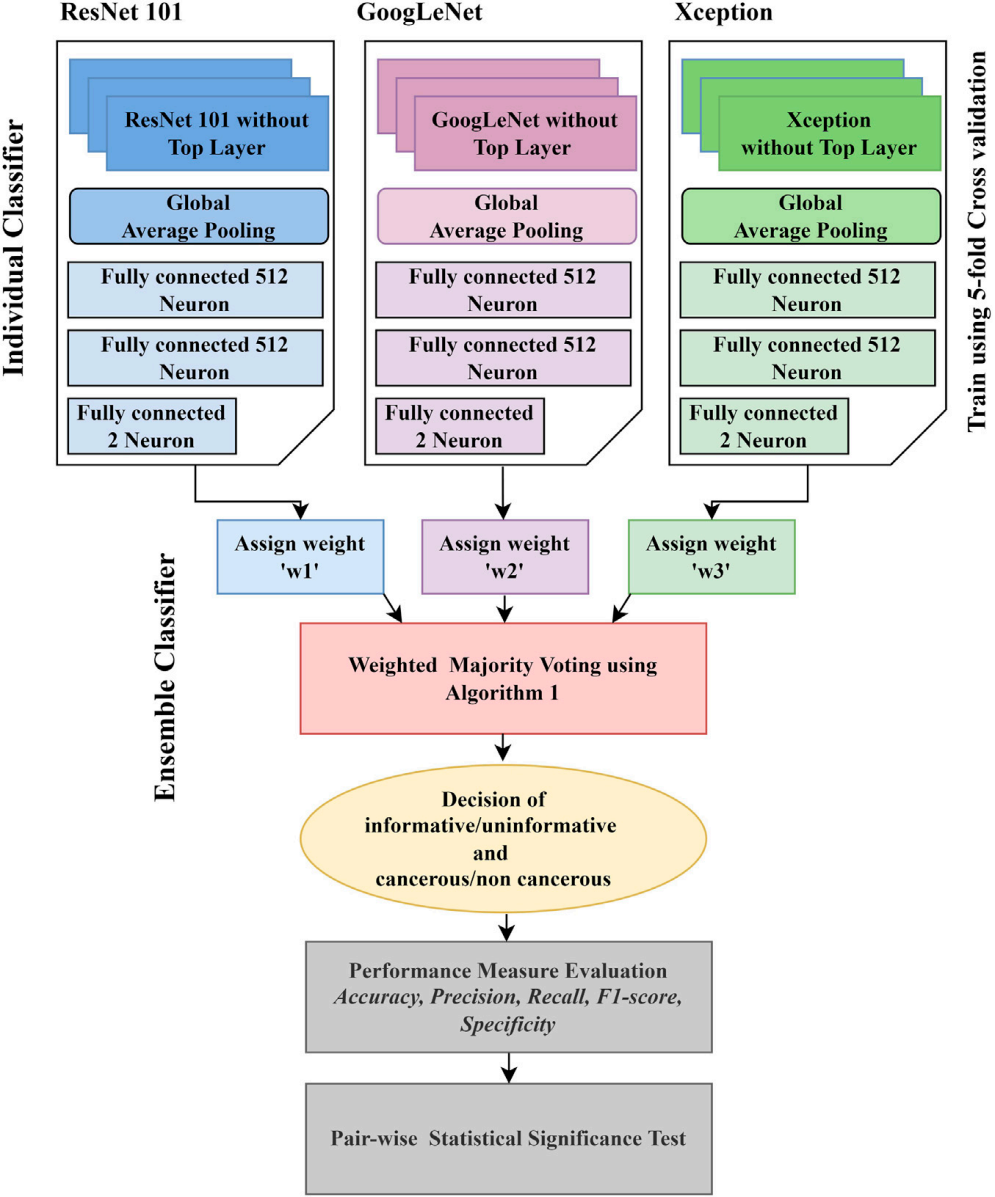
Workflow of the proposed system.

Conventional techniques for classification tasks rely on manually examined features. Optimal feature selection plays a vital role in the final outcome of the selected computer vision task. Identifying the best features for a target segmentation/classification algorithm is difficult due to less intergroup variability. The variability in the visual appearance of polyps and their background is much less compared to the object and its background in natural images. Therefore, algorithms that are efficient for computer vision task in natural images are not always an ideal approach to deal with the computer vision task in medical imaging. Deep learning is an active domain in the research area of medical image analysis as it has recently successfully overcome the challenges in image recognition on the ImageNet dataset (Deng et al., 2009). Hence, the application of the CNN, a deep learning approach in CRC analysis, is introduced in this work. The workflow of the proposed work is described in Figure 1.

### 2.1 Dataset

A dataset that consists of colonoscopy frames extracted from a colonoscopy video is used in this work. The data were generated in the Department of Gastroenterology, Aichi Medical University, Nagakute, Japan, with the IRB approval of the Aichi Medical University ethical committee (15 January 2018; Approval No. 2017-H304). To assess the robustness of the proposed methodology, evaluation is performed on two publicly available benchmark datasets, Kvasir (Pogorelov et al., 2017) and Depeca (Mesejo et al., 2016). The mentioned datasets can be downloaded from https://datasets.simula.no/kvasir/and http://www.depeca.uah.es/colonoscopy_dataset/, respectively. The details of these datasets are summarized in Table 1. Because of the unavailability of any polyp dataset that contains only two groups, that is, cancerous and noncancerous, we combine serrated and adenoma frames available on the Depeca colonoscopy dataset, which has a total of 55 original frames. We consider this combined class as cancerous and 21 hyperplastic frames as noncancerous in our study.

**TABLE 1:** Summary of datasets used in this study.

Dataset	# Frames		# Frames with polyps	
	Informative	Uninformative	Cancerous	Noncancerous
Aichi-Medical dataset	397	500	125	272
Kvasir dataset	500	500	-	-
Depeca colonoscopy dataset	—	—	55	21

### 2.2 Classification Model

The CNN typically requires a massive dataset for training (at least thousands of samples if not available in millions). Thus, the application of the CNN trained from scratch is difficult because limited time and workload of experts to create labeled sample datasets on medical images. If the available training dataset is small in size, as is the case in this domain of medical image analysis, methods based on the CNN usually overfit and are unable to extract the image features in high quality.

Transfer learning is the strategy through which a CNN is initially trained to learn standardized image characteristics on a large-scale labeled image dataset and then used to retrieve similar features from a smaller dataset. It has already been successfully applied in different image analysis tasks or disease-related trials. Therefore, in our proposed Ensemble classifier, the base model weights are transfer-learned from the ImageNet dataset (Deng et al., 2009). Data augmentation is applied to all the datasets for balancing the dataset. The augmentation techniques such as shearing, rotation, skewing, zooming, and inverting are used. It is one of the most common approaches used for minimizing overfitting during the training phase of the CNN. This approach artificially expands the dataset using different class-preserving functions applied to each image to generate synthetic images. The concept behind the augmentation techniques is that the reproduced samples do not change their semantic meaning but enable the generation of a new sample to increase dataset size. As mentioned earlier, training CNNs on large data leads to improvement in its efficiency, robustness, and generalizability on previously unseen data or samples. Hence, in this work, we apply clock-wise rotation with an angle of 45°, 90°, and 120° and zooming parameters of 30.00 and 10.00% to the 1,000 original images of the Kvasir dataset to generate another 1,000 augmented images. Due to fewer data in the Aichi-Medical dataset, we apply a shearing operation with a value of 0.1 to original frames and the augmentation as mentioned above to balance the class disparity in the number of images. Again, for the Depeca colonoscopy dataset, we apply rotation, shearing, inverting, skewing, and zooming to obtain a total of 2000 images, including the original image. After applying augmentation, each individual dataset contains 2000 images. Then, a two-level classification is carried out in this research to fulfill the objective.

• The first-level classification is for informative frame detection. The outcome of the classifier is expected to be the class label of individual frames as informative or uninformative.
• The second classification is to detect cancerous polyps from informative frames. The outcome of the classifier is expected to be the class label of an individual informative frame as a cancerous or noncancerous polyp.

Three CNN architectures are used along with the proposed Ensemble classifier. The description of individual classifiers is widely available in the literature.

• ResNet101: As the information from the input or the gradient calculated by the CNN passes through many layers, it sometimes vanishes in between the hidden layers and sometimes rinsed out by the time it hits the end or beginning of the network (Simonyan and Zisserman, 2014; Huang et al., 2017). This was solved using ResNet. Conventional neural networks forward the output information of a layer (e.g., *Lth*) as an input to the successive layers (*L* + 1)^*th*^. If X is the input to the *Lth* layer, then the input to the *L* + 1st layer will be *X*′, where *X*′ can be represented as

$$X^{\prime }=f\left(X\right).$$ (1)

Here, *f* is the series of different operations within the convolution block. ResNets have added a skip-connection that bypasses the nonlinear transformations with an identity function (Szegedy et al., 2015)

$$X^{\prime }=f\left(X\right)+X.$$ (2)

In this structure, input images are convolved by a kernel of size 7 × 7 with a stride equal to two followed by max-pooling. The first residual block accepts the output of this pooling layer. It uses a residual connection that adds the output of the pooling layer with the output of the first residual block. The residual block is constituted of three subsequent convolution layers. The first and third convolution operations are 1 × 1 convolution. The first convolution mixes up all the local properties of the image pixels across all the channels, and the convolution layer with 3 × 3 kernel mixes up the spatial properties. The third convolution layer helps increase the number of channels. The residual connection does not have any attenuation or gradient multiplication with activation. So, it is a unity gradient. By virtue of a residual connection, the exact value of the gradient can propagate back to the input layer. Using this structure, it is possible to carry forward information to the end of the model, but it is possible to backpropagate the gradient without vanishing it. The main power of ResNet is the direct flow of gradient through the identity relation from the successive layers to the prior layers.

• GoogLeNet: In the GoogLeNet architecture, a new “Inception” subnetwork module is added. The findings of various parallel convolution filters present at the inception are concatenated. The repetition of the Inception modules captures the optimal sparse representation of the image, while simultaneously reducing dimensionality. The network comprises 22 layers that require training (or 27 if pooling layers). Experiments have shown that GoogLeNet has fewer trainable weights than AlexNet and, thus, is more accurate (Szegedy et al., 2015).
• Xception: In the structure of Xception, the convolution layer used in ResNet is replaced by a depth-wise separable convolution module. Depth-wise separable convolution converges the process faster, and the accuracy is high. In the depth-wise separable convolution module, depth-wise convolution is followed by a 1x1 convolution. The number of filters is equal to the number of channels in each layer. With decreasing number of channels, the number of connections also decrease, which eliminates the drawback of performing convolution across all the channels. The depth-wise separable convolution learns spatial correlation, and the 1x1 convolution learns the interchannel correlation. The nonlinear activation function is not used. As state-of-the-art literature conveys that Xception outperforms VGG-16 and ResNet-152 in the ImageNet classification challenge (Chollet, 2017), Xception retains the characteristics of ResNet and can effectively deal with the complex situation of extracting targets covered by occlusions. Considering these advantages, in our proposed Ensemble method, we used Xception as a candidate model that is optimized based on ResNet.

Each individual classifier is fine-tuned according to our objective. Because the classification task in this work is to deal with binary classification problems, the models are fine-tuned by truncating the top layers of each model and replacing them with a modified fully connected network with a two-neuron output layer. Finding the best model for a specific task is dependent on efficient hyperparameter optimization. The best hyperparameters considered in this work are Adam as an optimizer, 0.001 learning rate, and a batch size of 32.

### 2.3 Ensemble Classifier

Ensemble classification is the preference of many scientists in a variety of fields such as computer vision and medical image analysis. For example, Bolón-Canedo et al. (2012) developed an Ensemble classification approach in the bioinformatics field, aiming for interpretation of the microarray data classification. Sun et al. (2015) implemented the concept of Ensemble classification on an imbalanced dataset. They reported that it outperformed conventional classification techniques. Sharif et al. (2020) applied an Ensemble classifier to analyze the data for squamous cell carcinoma. Bose et al. (2021) proposed an Ensemble classifier for efficient classification of a malignant tumor. Shakeel et al. (2020) used an Ensemble classifier to detect non–small cell lung cancer from CT images. Hussain et al. (2020) also used the Ensemble classifier to mine the data in cervical precancerous samples and cancer lesions. Some other biomedical research contributions based on the application of the Ensemble classifier to improve computer-aided systems can be found in references (Yang et al., 2016; Yang et al., 2020; Ayaz et al., 2021; Mahfouz et al., 2021). These ensembles are basically combining traditional machine learning models, such as SVM and AdaBoost, with one of the deep learning models. Our motivation for this work is to find a novel and efficient model for classifying colonic polyps to detect colorectal cancer. So far, there has been limited work that focuses on improving the performance of polyp detection using Ensemble. This encouraged us to incorporate the principle of Ensemble classification in this work. In the Ensemble method, the approach is to consult as many classifiers as possible and factor their decision in such a way that its efficiency will be enhanced. Figure 1 presents an overview of the proposed Ensemble method. Unlike most other ensembles in the literature, which rely on handcrafted features, we use three of the best performing CNN models in both the computer vision and medical imaging tasks in our Ensemble. Initially, the CNN architectures whose weights have been initialized on natural image data are fine-tuned. Each of the fine-tuned CNN extracts independent image features to classify an image. Then, the Ensemble classifier chooses the class label for a particular image based on the decision of each candidate classifier. To consider the decision of each individual classifier, a weight is assigned to each individual decision based on the weighted majority voting technique. The process of decision making by the Ensemble classifier is detailed in Algorithm 1. During the decision-making process, we consider the loss of each individual model when deciding the class-label probability for each image. The individual model that has the smallest loss will be assigned the highest weight.

Algorithm 1 Decision of the Ensemble classifier.

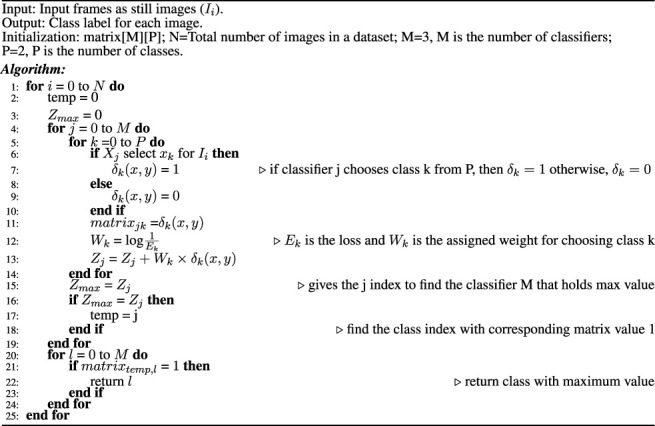

### 2.4 Performance Metrics for Evaluation of Classification Task

The performance evaluation parameters of a classification model are based on the correct and incorrect estimation of test records anticipated by the model. The confusion matrix gives the insight of predicated values compared to the actual values that can be visualized for the test dataset for all the classes. The four measures, true positive (TP), false positive (FP), true negative (TN), and false negative (FN), are part of the confusion matrix. Based on these four measures, efficient parameters to evaluate different classification techniques can be estimated. The most common performance measures based on the confusion matrix are explained in Table 2. This work has considered accuracy, precision, recall, F1 score, and specificity to evaluate the performance of our Ensemble classifier.

**TABLE 2:** Performance measures for evaluating the detection model.

Measures	Formula	Description
Accuracy Urban et al. (2018); Zhang et al. (2016); Bandyopadhyay et al. (2013); Bedrikovetski et al. (2021)	$\frac{\left|TP\right|+\left|TN\right|}{\left|TP\right|+\left|TN\right|+\left|FP\right|+\left|FN\right|}$	The ratio of the number of correct prediction with respect to total observations
Precision Urban et al. (2018); Zhang et al. (2016); Bandyopadhyay et al. (2013); Bedrikovetski et al. (2021)	$\frac{\left|TP\right|}{\left|TP\right|+\left|FP\right|}$	The ratio of the number of correct positive prediction with respect to total positive prediction
Recall/Sensitivity Urban et al. (2018); Zhang et al. (2016); Bandyopadhyay et al. (2013); Bedrikovetski et al. (2021)	$\frac{\left|TP\right|}{\left|TP\right|+\left|FN\right|}$	The ratio of number of correct positive prediction with respect to actual positive observation
F1 score/Dice-coefficient Urban et al. (2018); Zhang et al. (2016); Bandyopadhyay et al. (2013); Bedrikovetski et al. (2021)	$2\times \frac{Recall\times Precision}{Recall+Precision}$	F1 score is the harmonic mean of both precision and recall

### 2.5 Statistical Analysis

The statistical significance test is applied to compare the significance of our proposed Ensemble method with others. We used the McNemar test (McNemar, 1947; Demšar, 2006) with a contingency table. The McNemar test is used to compare the accuracy of prediction for two models.

## 3 Results and Discussion

To evaluate the efficiency of the proposed method and compare their performance with the existing methods, we applied and evaluated the proposed Ensemble method along with the individual classifier on our generated dataset. These models were implemented using the Keras deep learning framework with a TensorFlow backend provided by Google-Colab.

**FIGURE 2:**
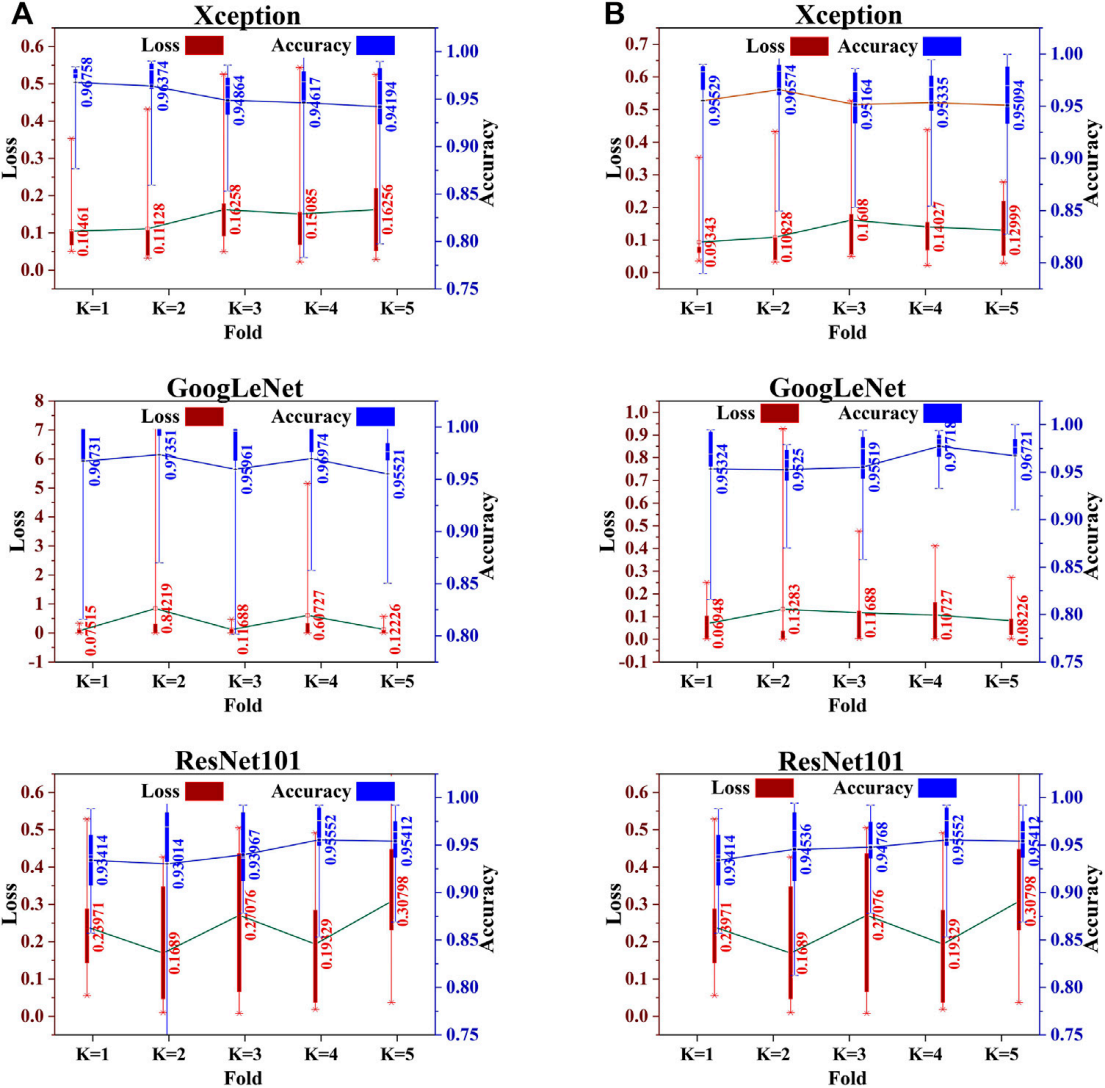
Five-fold cross-validation accuracy and loss of each individual classifier for **(A)** informative frame detection and **(B)** cancerous and noncancerous polyp categorization.

The dataset was split into two subsets using the train–test strategy. We first consider splitting with a ratio of 0.15. The first subset of 300 images is considered only for testing model performance, while the second subset of 1700 images is used for training. In the training phase, five-fold cross-validation is applied wherein each fold with 15% of the training data is considered for validation to improve the performance of the model. We train the same base classifier individually for each classification task to achieve both the objectives of this work. First, we perform the classification of informative and uninformative frames. Then, we conduct a separate training for all three classifiers for the second classification purpose, that is, to classify cancerous and noncancerous polyps. The box and whisker plots in Figures 2A,B show the mean score of the validation accuracy and loss achieved during each fold for each individual classifier. GoogLeNet achieved 96.5% average accuracy after five-fold cross-validation, which is the highest among all three individual classifiers for both classification tasks. For the test dataset, the accuracy, precision, recall, F1 score, and specificity values were reported for all the classifiers.

### 3.1 Evaluation of Classifier Performance

**FIGURE 3:**
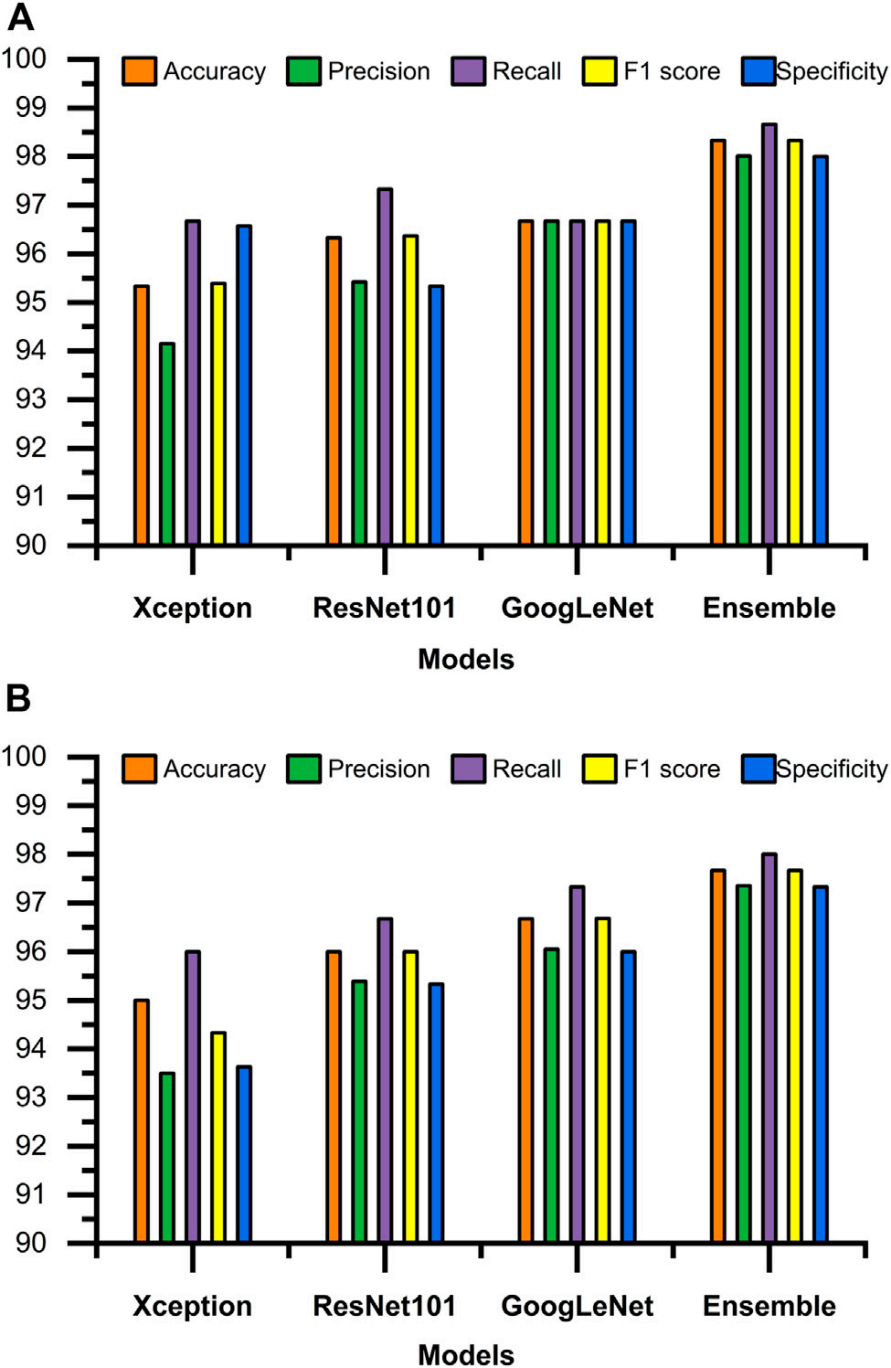
Test results of all four classifiers for **(A)** informative frame detection and **(B)** cancerous and noncancerous polyp classification.

**FIGURE 4:**
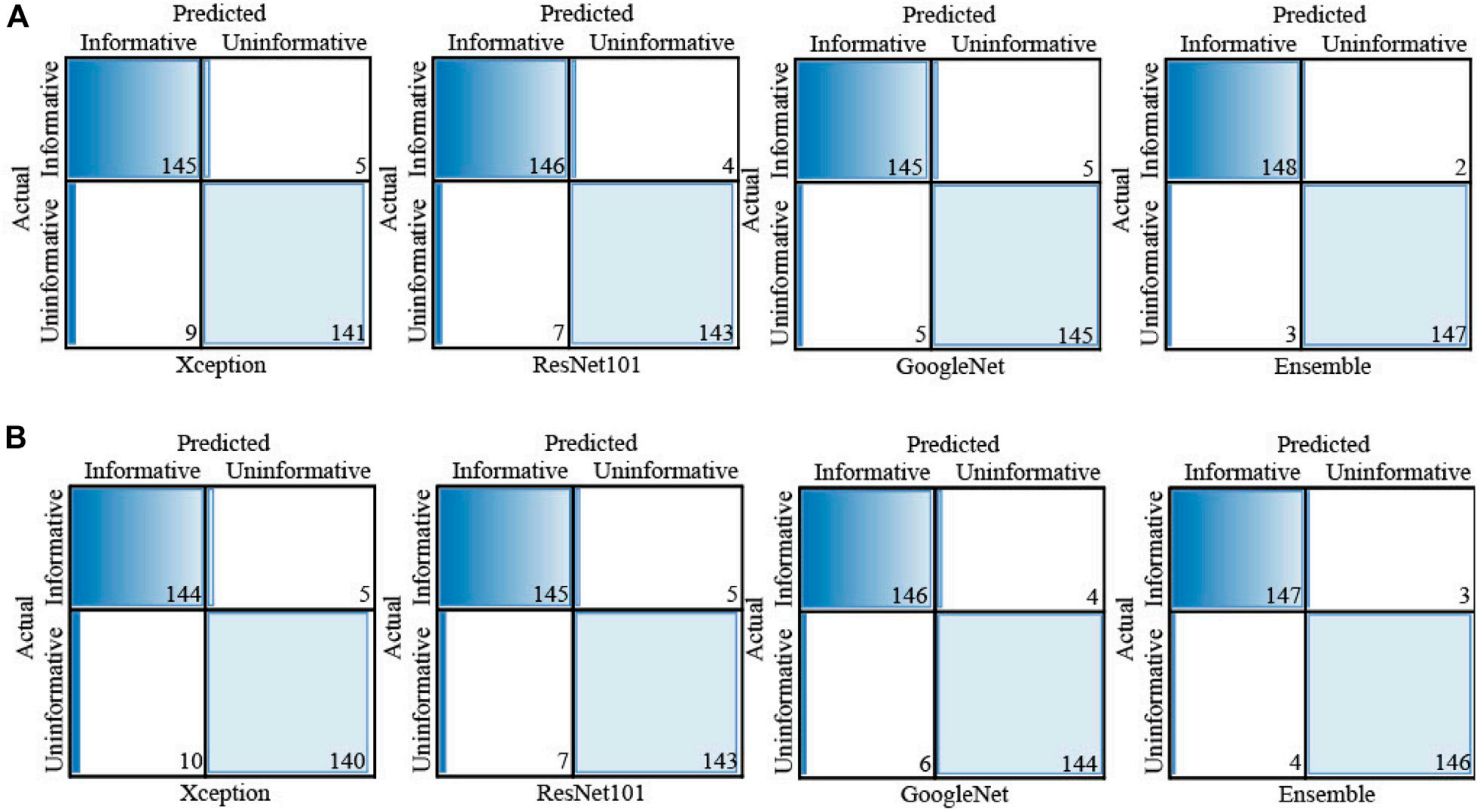
Confusion matrix of each individual classifier for **(A)** informative frame detection and **(B)** cancerous and noncancerous polyp classification.

**FIGURE 5:**
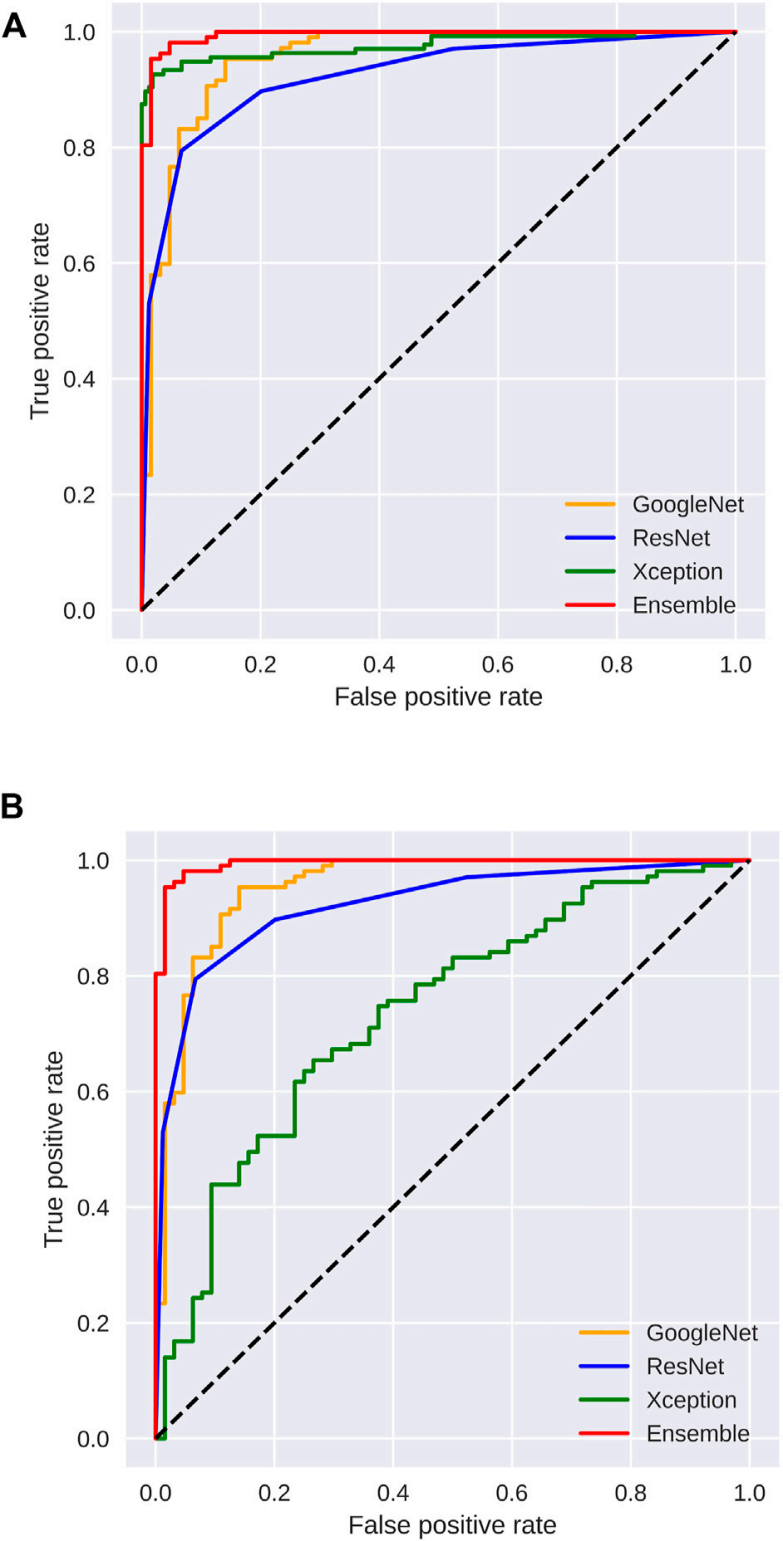
Area under the ROC curve analysis for **(A)** informative frame detection and **(B)** cancerous and noncancerous polyp classification.

Figures 3, 4 display the performance of the Ensemble classifier along with each individual classifier on the generated dataset. For informative frame detection, our proposed Ensemble obtained 98.3, 98.6, and 98.01% accuracy, precision, and recall, respectively, and for cancerous polyp detection, 97.66, 98.66, and 96.73% accuracy, precision, and recall, respectively. We performed receiver operating characteristic (ROC) analysis, and Figure 5 shows the model performance using the measured area under the ROC curve (AUC). These observed results indicated that our proposed Ensemble classifier performed better than any other classifiers for both classification tasks.

Based on our objective of this work, both the FP and FN are crucial, and our goal is to keep them low. In the first scenario, our proposed system informs that patients having a cancerous tumor but being labeled as noncancerous could lead to misclassification denoted as false negative (FN). In another scenario, patients not having a cancerous tumor but being informed as abnormal (cancerous) could cause false positive (FP). Both FNs and FPs have a significant impact on misclassification, therefore leading to wrong diagnosis and causing human health problems. We considered F1 score along with other performance evaluation measures to equally prioritize both FP and FN. We observed that our proposed method gives the highest F1 score of 98% for informative frame detection and 97.33% for cancerous polyp detection. Almost equal precision, recall, and F1 score of our ensemble convey that the proposed model has a negligible rate of misclassification, which is also supported by the specificity value.

**FIGURE 6:**
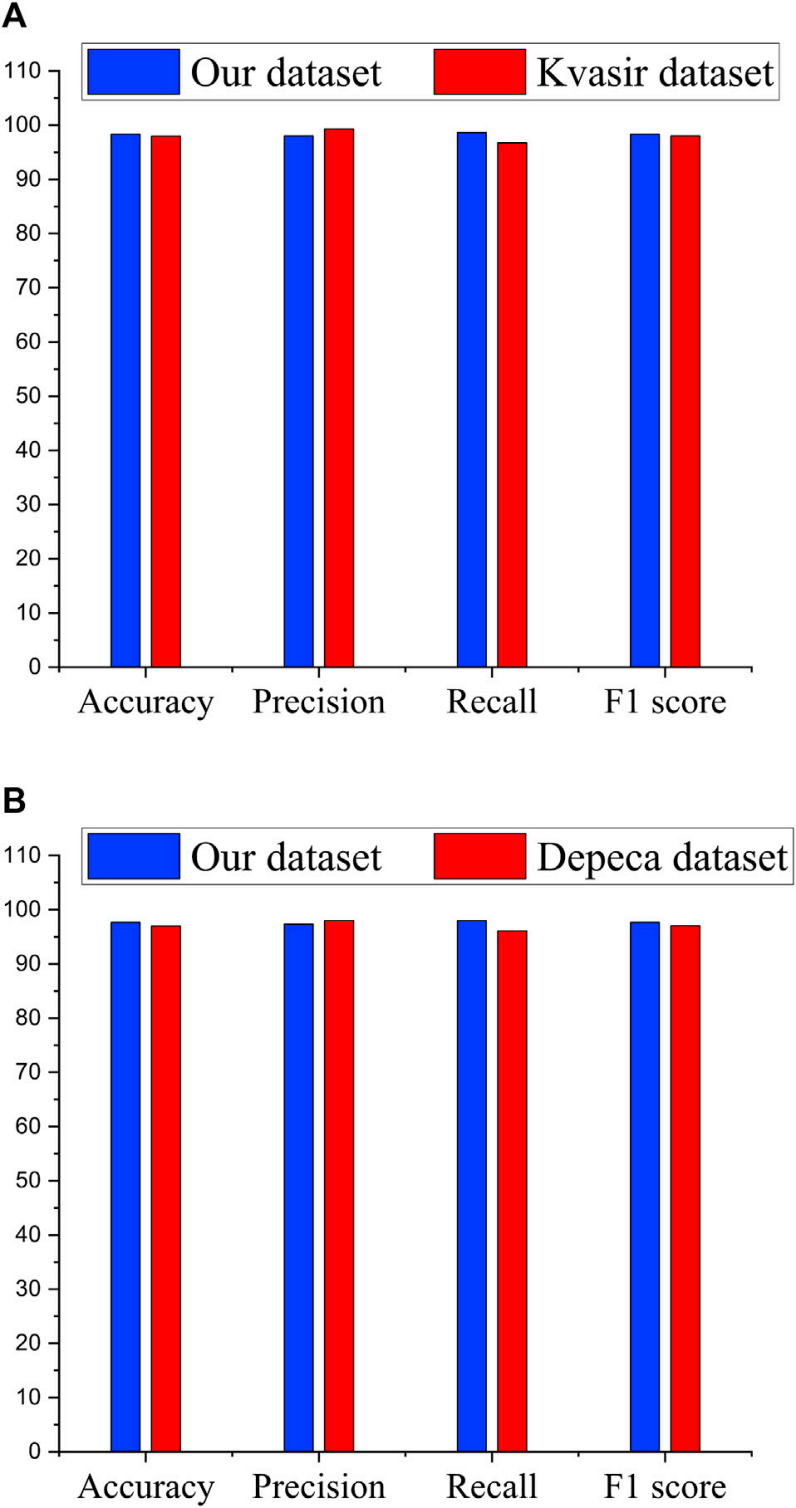
Performance comparison of Ensemble classifiers. **(A)** Performance of Ensemble classifier on significant frame detection. **(B)** Performance of Ensemble classifier on classification of cancerous and noncancerous polyps.

Figure 6A shows the comparison of our Ensemble classifier’s result on the Kvasir dataset with our dataset. The Kvasir dataset is considered a benchmark dataset for informative frame detection, and our Ensemble attains a value of test accuracy 98%, precision 99.33%, recall 96.75%, F1 score 98.03%, and specificity 99.31%. Figure 6B compares the Ensemble classifier’s result on the Depeca colonoscopy dataset to produce the effectiveness of our proposed method on a new independent dataset for cancerous polyp detection. We observed that the results were consistent on all the datasets, which provides clear evidence of the robustness of our proposed method.

In Table 3, we compared the Ensemble classifier with other classifiers found in existing literature for CRC detection (Zhang et al., 2016; Shin and Balasingham, 2017; Akbari et al., 2018; Urban et al., 2018; Wang et al., 2018; Sharma et al., 2020a). From the observation, it is comprehendible that the proposed method outperforms the other classifier and is efficient in fulfilling our objective.

**TABLE 3:** Classification performance in comparison with similar work.

Objective	Methods	Algorithm	Accuracy	Precision	Recall	F1 Score	Specificity
Informative frame detection	Proposed Ensemble	CNN	**98.3**	98.6	**98.01**	**98.33**	98.66
	Akbari et al. (2018)	CNN	90.28	74.34	68.32	71.20	94.97
	Zhang et al. (2016)	Ensemble (SVM + CNN)	98.0	**99.4**	97.6	98.00	-
	Shin and Balasingham (2017)	CNN	86.69	86.28	28.90	43.30	99.02
	Liew et al. (2021)	Ensemble (ResNet50 + Adaboost)	97.91	99.35	96.45	—	**99.38**
Cancerous and noncancerous polyp identification	Proposed Ensemble	CNN	**97.66**	**98.66**	**96.73**	**97.68**	**98.63**
	Urban et al. (2018)	CNN	90.00	—	88.1	—	—
	Zhang et al. (2016)	Ensemble (SVM + CNN)	85.90	87.30	87.60	87.00	—
	Wang et al. (2018)	CNN	90.00	—	94.50	—	—
	Patino-Barrientos et al. (2020)	CNN	83.00	81.00	86.00	83.00	—

*Bold values indicate the best performance.

In aid of this interpretation, the McNemar test result of our Ensemble paired with each individual classifier is summarized in Table 4. The *p*-values obtained from this test are less than 0.05 in all the cases. These results show that our proposed Ensemble approach is superior to the other classifiers.

**TABLE 4:** Significance of Ensemble classifier decision in comparison with individual classifiers.

Classifier	Chi-squared Value	*p*-Value*
ResNet101 vs. Ensemble	4.16	0.041
GoogLeNet vs. Ensemble	2.28	0.039
Xception vs. Ensemble	6.75	0.009
ReNet101 vs. Ensemble	2.25	0.033
GoogLeNet vs. Ensemble	2.25	0.033
Xception vs. Ensemble	5.81	0.015

* *p*-value is based on the McNemar test.

### 3.2 Discussion

Based on the results, it is confirmed that the proposed Ensemble classifier is an efficient model for colonoscopy image analysis and can be used as an assistant tool by the gastroenterologist during the screening of CRC. Even though the proposed method shows better performance, some clinical information such as sex and age of the patients, other medical conditions, geographic location, etc. are not considered in this work. Future work conducted by considering these criteria can improve computer-aided systems for early cancer detection and treatment in personalized medicine. As the massive dataset available for transfer learning contains natural images, the transfer-learned features are more reflective of the natural image characteristics and may not always necessarily reflect the subtle characteristics of medical images. Therefore, it is expected that transfer learning from the same domain large-scale dataset will lead to developing a more efficient automatic system for CRC analysis. Kudo et al. (1996) has reported that the detection rate to differentiate cancerous and noncancerous lesions using images from magnifying endoscopy is higher (81.5%) than that of the stereomicroscopic analysis. Therefore, a performance comparison of the proposed model considering the images of magnifying endoscopy and the colonoscopy images will be a future direction.

## 4 Conclusion

In this article, we introduced a new Ensemble method for the classification of each individual frame of a colonoscopy video as informative or uninformative and then for predicting the classified informative frames as cancerous or noncancerous polyps. Our Ensemble uses multiple fine-tuned CNNs that can learn diverse information present in individual images. The Ensemble can fuse the fine-tuned CNN models to derive a more powerful image classification scheme than the individual CNNs. When Xception extracts features, it achieves the best performance because Xception is optimized on the basis of ResNet, which makes Xception inherit not only ResNet’s advantage of residual connection but also its ability to extract objects when covered by occlusions through depth-wise separable convolution. The analysis by the McNemar statistical test indicates high significance in the performance of the Ensemble classifier when compared to the individual classifiers. Therefore, our Ensemble shows the best performance for polyp detection on colonoscopy with an acceptable level of all performance measures in the range 0.95–1. A minor difference in precision and recall value of our Ensemble classifier indicates that it can accurately detect the presence of a polyp and also differentiate the cancerous from noncancerous polyps efficiently.

## Data Availability

The datasets presented in this article are not readily available due to ethical restrictions; we cannot publish the data currently. If required, we can provide the sample dataset after acceptance. Requests to access the datasets should be directed to KB, kangkana.bora@cottonuniversity.ac.in.
